# Susceptibility of Livestock, Wildlife, and Domestic Host Species Cells to the 2022–2025 Reassortant Oropouche Virus

**DOI:** 10.3390/pathogens15040367

**Published:** 2026-03-31

**Authors:** Lindsey M. Reister-Hendricks, Dane C. Jasperson, Jessica Gutierrez, Bethany L. McGregor, Stacey L. P. Scroggs

**Affiliations:** 1 Arthropod-Borne Animal Diseases Research Unit, Agricultural Research Service, United States Department of Agriculture, Manhattan, KS 66502, USA; 2 Center for Grain and Animal Health Research, Agricultural Research Service, United States Department of Agriculture, Manhattan, KS 66502, USA; 3 Department of Agricultural Resource Management, Wisconsin Department of Agriculture, Trade, and Consumer Protection, Madison, WI 53708, USA

**Keywords:** OROV, *Orthobunyavirus*, arbovirus, host susceptibility, wildlife, livestock, animal, cell culture, virus replication curve

## Abstract

Oropouche virus (OROV) is an emerging zoonotic arthropod-borne virus of public health importance. The host range of OROV is largely unknown, but antibody evidence suggests that wildlife and livestock species could be susceptible hosts. To identify potential North American mammalian reservoir hosts, OROV replication curves were generated using eight cell lines derived from livestock, wildlife, and domestic animal species (cow, sheep, bison, white-tailed deer, elk, pig, horse, and dog). The virus replicated in all cell lines by 48 h post infection, except for the horse cells. OROV replication success was greatest in the bison cells followed by pig and dog cells. Moderate replication was achieved in the deer, elk, sheep, and cow cells. These results indicate that numerous animal species may be susceptible hosts for OROV, including important agricultural and wildlife species, but pathogenesis studies are required to confirm this finding. Identifying the reservoir hosts for OROV will allow livestock producers, veterinarians, and public health officials to prepare appropriate vector and disease control measures should the virus initiate an outbreak in the United States.

## 1. Introduction

Oropouche virus (OROV, Peribunyaviridae, *Orthobunyavirus oropoucheense*) is an emerging zoonotic arthropod-borne virus in the Americas that causes Oropouche fever, a febrile illness in humans [1,2]. The virus was originally isolated in 1955 from a febrile man in Trinidad and Tobago [3] and is now considered endemic throughout the Amazonian region with particularly high incidence in Brazil [4]. Historically, mortality was not recorded from OROV outbreaks [5], although incidence of OROV is thought to have been underreported due to its similarity in presentation to other circulating febrile illnesses, such as dengue [6]. In 2022, a large-scale, multi-country outbreak spread to non-endemic regions as far north as Cuba [7] with over 29,000 suspected cases [8] and 110 travel-associated cases in the United States [9]. The ongoing outbreak also represented the first documented cases of OROV mortality and vertical transmission to an unborn fetus [10].

This outbreak was precipitated in part by characteristics of the OROV genome. OROV has a tri-segmented, single-stranded, negative-sense RNA genome, which is highly susceptible to genetic reassortment during co-infection and the emergence of novel reassortant viruses [11]. OROV reassortants have emerged from the endemic region multiple times, including the emergence of Iquitos virus, Madre de Dios virus, Perdões virus, and the 2022–2025 OROV (denoted from here on as OROV^240023^). The novel reassortant OROV^240023^ has been shown to have enhanced replication in both mammalian cells [12] and within the vector [13], resulting in the largest recognized OROV outbreak to date.

OROV is transmitted by species of *Culicoides* biting midges (Diptera: Ceratopogonidae), particularly *Culicoides paraensis* within its endemic range [14]. These small flies (1–5 mm) feed on blood in order to develop eggs, a behavior through which they can transmit numerous viruses of veterinary importance, including bluetongue virus, epizootic hemorrhagic disease virus, vesicular stomatitis virus, African horse sickness virus, and Schmallenberg virus [15,16]. Sylvatic OROV is likely maintained between biting midges and sloths and primates [17], but antibody evidence suggests that wildlife and livestock species, including cattle [18], are exposed to OROV and could be susceptible hosts [19,20]. Identifying susceptible animal hosts and reservoirs for OROV is essential for understanding the transmission dynamics of OROV, especially as the virus emerges into new geographic locations.

To identify whether OROV poses a threat to North American livestock, wildlife, or domestic pets, we used an ex vivo model to investigate the susceptibility of a variety of mammalian hosts to OROV infection. We found that lung and/or kidney cells isolated from cows, sheep, bison, white-tailed deer, elk, pigs, and dogs were susceptible to OROV^240023^ infection as evidenced by increased viral titers above the initial inoculum. Horse-derived cells did not support OROV replication. Based on cell culture infections, bison, pigs, and dogs appear to be at the highest risk for OROV infection, but in vivo experiments are needed to confirm these findings and determine pathogenicity.

## 2. Materials and Methods

### 2.1. Cells and Virus

The cells used in this study are listed in Table 1. Species were selected for inclusion based on their potential to serve as animal reservoirs for OROV in North America. Primary lung or kidney cells isolated from the following domestic and wild ruminant species in the United States were included: *Bos taurus* (cow), *Ovis aries* (sheep), *Bison bison* (American bison)*, Odocoileus virginianus* (white-tailed deer)*,* and *Cervus canadensis* (elk). Kidney cells from the non-ruminant *Sus scrofa* (pig) and from two domestic, non-livestock species (*Equus caballus* (horse) and *Canis lupus familiaris* (dog) were included based on their agricultural importance (pig), population numbers (pig, horse, and dog), close contact with livestock species (horse and dog), antibody evidence (dog), and availability. All primary cell lines were initiated and established by the USDA Insectary and Cell Culture Laboratory (ICCL) from tissues derived from specimens donated to the Arthropod-Borne Animal Diseases Research Unit (ABADRU) or obtained from the Wyoming State Veterinary Diagnostic Laboratory (WSVDL). Briefly, primary lung and kidney cells were harvested by manual homogenization and then pressed through tissue sieves. Subsequent cells were washed and resuspended in Medium 199 supplemented with 20% fetal bovine serum (FBS) and 1× antibiotic–antimycotic. Non-adherent cells were removed and media replaced at weekly intervals. Cell lines were screened for bacteria, fungi, and mycoplasma contamination by appropriate cultivation methods. Specific tests for the presence of bovine viral diarrhea, bluetongue viruses and Brucella were also performed. Cell lines were additionally screened for other viral contaminants by electron microscopy using negative staining techniques. All cell lines used for this study were negative for contaminants included in the screening processes mentioned. The African green monkey Vero cell line (Vero MARU) is a clone from the Middle America Research Unit (MARU) and was donated to ABADRU. The MDCK (CCL-34) and SK-RST (CRL-2843) cells were obtained from the American Tissue Culture Collection (ATCC). All cells were grown at 37 °C with 5% CO_2_ in media supplemented with fetal bovine serum (FBS), 100 U penicillin/streptomycin sulfate, and 0.25 μg/mL of amphotericin B. The base media and percent serum are listed in Table 1 for each cell type.

The OROV^240023^ isolate was kindly provided by the Centers for Disease Control and Prevention. OROV^240023^ was isolated from a travel-associated case of OROV that was acquired in Cuba in 2024 then diagnosed in Florida. The virus was passaged three times in Vero cells followed by ultracentrifugation and storage at −80 °C. The RNA sequence of the OROV stock was confirmed by whole-genome, long-read sequencing. RNA was reverse-transcribed and amplified using the SuperScript™ IV One-Step RT-PCR System (Thermo Fisher Scientific, Waltham, MA, USA) per manufacture instructions with OROV specific primers [21]. Libraries were prepared using Rapid Barcoding Kit V14 (Oxford Nanopore Technologies Inc., Oxford, UK) and sequenced on a MinION platform (Oxford Nanopore Technologies Inc., Oxford, UK) to confirm 99.97%, 99.98%, and 99.86% identify with the L, M, and S segments of the original isolate (PQ417948-50). Compared to the original isolate, two non-synonymous variants are present in the L segment (F63L) and M segment (K748E) and two silent variants are present (one each in segments L and S).

### 2.2. Multi-Cycle OROV Replication Curves

Triplicate T25 flasks of each cell type were infected with OROV^240023^ at a multiplicity of infection (MOI) of 1.0 followed by a 2 h incubation at 37 °C with gentle rocking every 20 min. The MOI was calculated using the OROV viral titer determined in Vero cells. After 2 hr, the inoculum was removed and the cells were washed twice with 1× phosphate-buffered saline (PBS). The last wash was collected for each flask as the 0 hr post-infection timepoint (HPI). After washing, 5 mL of media was added to each flask, and the flasks were incubated at 37 °C with 5% CO_2_. At 12, 24, and 48 HPI, 1 mL of viral supernatant was removed from each flask, clarified by centrifugation at 1200 rpm for 10 min at 4 °C and stored at −80 °C. Immediately after the media was removed, 1 mL of fresh media warmed to 37 °C was added to each flask to keep the 5 mL total volume.

Infectious virus was quantified via plaque assay. Vero MARU cells were seeded in 6-well plates at 1 × 10^6^ cells per well and allowed to reach confluency. Samples were diluted 10-fold in cell culture media, then 200 μL was inoculated onto the cell monolayers in duplicate. Plates were incubated at 37 °C for 2 hr with gentle rocking every 20 min then overlaid with 1% methylcellulose (4000 cp.) in supplemented 199e media. The plates were incubated at 37 °C with 5% CO_2_ for 3 d then fixed and stained with 1% crystal violet, 25% formaldehyde to visualize and count plaques.

Viral RNA was extracted for each species and timepoint using the KingFisher Apex nucleic acid extraction system (Thermo Fisher Scientific, Waltham, MA, USA) per manufacturer protocols. Ct values were obtained in triplicate via RT-qPCR with the Applied Biosystems 7500 Fast real-time PCR system (Thermo Fisher Scientific, Waltham, MA, USA) using the TaqMan Fast Virus 1-step master mix (Thermo Fisher Scientific, Waltham, MA, USA) and 3 μl RNA template per well, as previously described [13,21]. RNA copies/mL was calculated using the slope (−3.35) and y-intercept (44.62) generated from a standard curve with an R^2^ of 0.99.

### 2.3. Statistical Analyses

To identify differences in viral replication within species, repeated-measures (RM) one-way ANOVAs with Tukey pairwise comparisons were used. To identify differences in viral replication between species, the percent change in viral titer was calculated using 0 HPI as the baseline followed by an RM two-way ANOVA. The area under the curves (AUC) was calculated by species and ranked from largest to smallest to estimate risk of host susceptibility to OROV infection. Statistics were conducted using Prism (v.10.4.1).

## 3. Results

### 3.1. OROV^240023^ Replicates Efficiently in Multiple Cell Lines Derived from Livestock, Wildlife, and Domestic Animals

**Figure 1:**
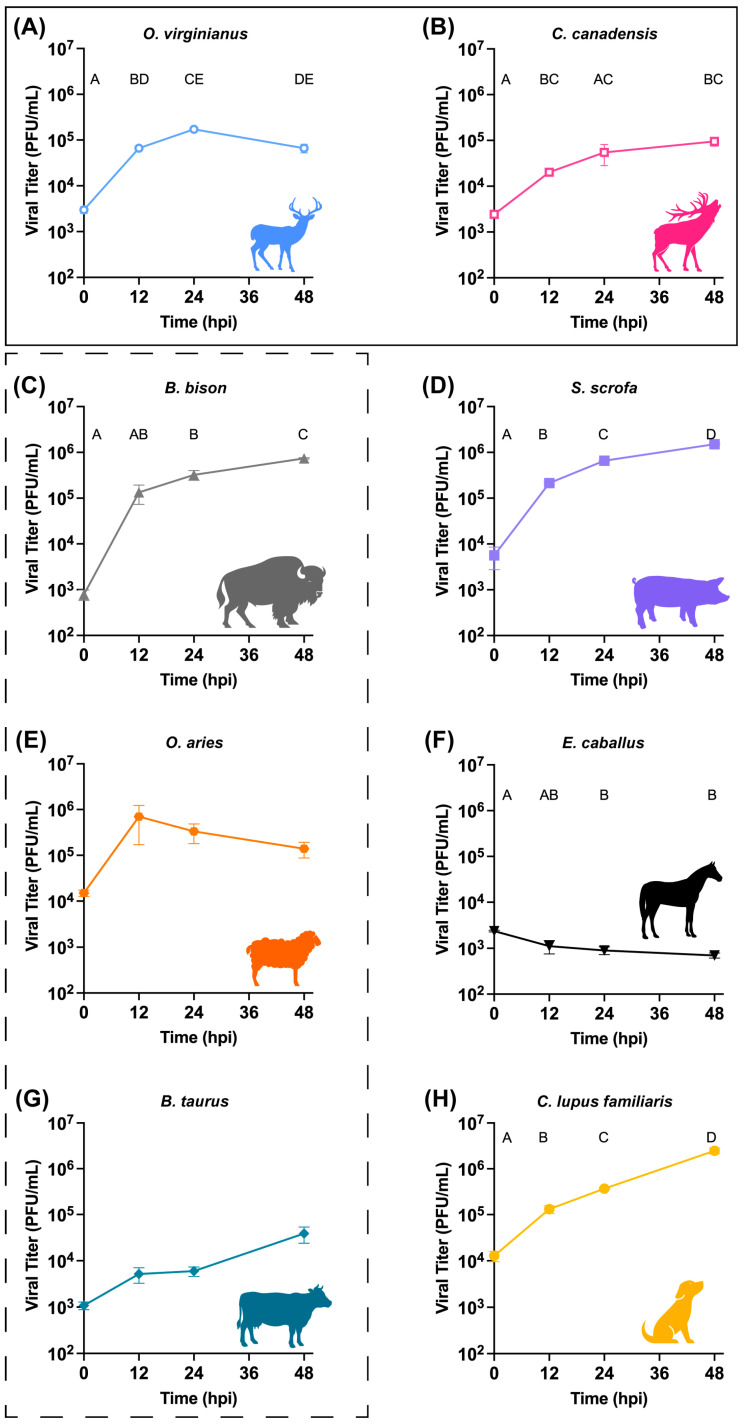
Multi-cycle replication curves of infectious OROV^240023^ in cells derived from eight host species, (**A**) *O. virginianus*, (**B**) *C. canadensis*, (**C**) *B. bison*, (**D**) *S. scrofa*, (**E**) *O. aries*, (**F**) *E. caballus*, (**G**) *B. taurus*, (**H**) *C. lupus familiaris*. Black solid box indicates Cervidae species. Black dashed box indicates Bovidae species. Virus detected at 0 HPI represents residual viral inoculum after washing the cell monolayer. Timepoints that do not share a letter are significantly different by Tukey pairwise comparisons (*p* < 0.05). The ANOVAs for panels without pairwise comparisons (**E**,**G**) were not significant. Full repeated-measures ANOVA results are reported in Table 2. The limit of detection is 50 PFU/mL.

Nearly all cell types tested supported OROV^240023^ replication. OROV^240023^ replicated in cells from both Cervidae species, *O. virginianus* and *C. canadensis* (Figure 1A,B), with viral titers peaking at 24 HPI for *O. virginianus* (mean 1.7 × 10^5^ PFU/mL) and plateauing after 12 HPI for *C. canadensis* (mean PFU/mL ranging from 2.0 × 10^4^ at 12 HPI to 9.5 × 10^4^ at 48 HPI).

In Bovidae species, OROV^240023^ replicated efficiently in *B. bison* cells and moderately in *O. aries* and *B. taurus* cells (Figure 1C,E,G). Viral replication in *B. bison* cells did not peak within the 48 hr timeframe of the experiment and the viral titer increased to an average of 7.4 × 10^5^ PFU/mL. Viral titers from the *O. aries* cells rose rapidly to peak at 12 HPI (7.0 × 10^5^ PFU/mL), but RM-ANOVA was not significant (Table 2). Replication in *B. taurus* cells increased slowly throughout the 48 hr, did not peak, and remained below 1.0 × 10^5^ PFU/mL, and the RM-ANOVA was also not significant (Table 2). OROV^240023^ replication in *S. scrofa* and *C. lupus familiaris* cells did not peak within the timeframe of the experiment and produced the highest viral titers by 48 HPI (*S. scrofa*: 1.5 × 10^6^ PFU/mL, *C. lupus familiaris*: 2.5 × 10^6^ PFU/mL) with over 10^6^ PFU/mL each (Figure 1D,H). In *E. caballus* cells, viral titers did not increase over time, indicating that viral replication did not occur (Figure 1F).

**Figure 2:**
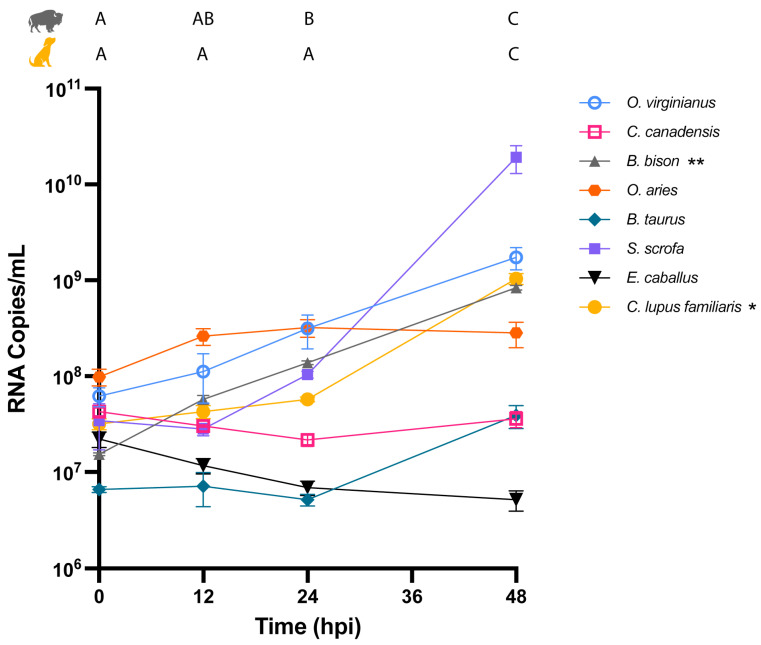
Time course of OROV^240023^ RNA copies after infection in cells from eight host species. Stars next to species names in the legend indicate significant RM-ANOVAs (* *p* < 0.05, ** *p* < 0.01). Tukey pairwise comparison results for *B. bison* and *C. lupus familiaris* are displayed above the graph. Timepoints that do not share a letter are significantly different by Tukey pairwise comparisons (*p* < 0.05). RNA copies/mL at 0 HPI represents residual RNA present after removing the inoculum and washing the cell monolayer. Full RM-ANOVA results are reported in Table 2. The limit of detection is 2.1 × 10^3^ copies/mL.

Viral S segment RNA produced in *B. bison*, *S. scrofa*, *C. lupus familiaris*, *O. virginianus*, and *B. taurus* cells increased over the time course, but the trends were only significant for *B. bison* and *C. lupus familiaris* (Figure 2, Table 2). Pairwise comparisons indicate there was significantly more viral RNA over the course of infection compared to earlier timepoints, reflecting increases of 1.74 log_10_ viral RNA copies/mL and 1.5 log_10_ viral RNA copies/mL from the time of inoculaion to 48 HPI for *B. bison* and *C. lupus familiaris,* respectively. Viral RNA produced in *O. aries* and *C. canadensis* remained unchanged over the course of infection, and it decreased in *E. caballus*, although the change was not significant (Figure 2, Table 2).

### 3.2. Susceptibility to OROV^240023^ Infection in Cells Is Species-Dependent

**Figure 3:**
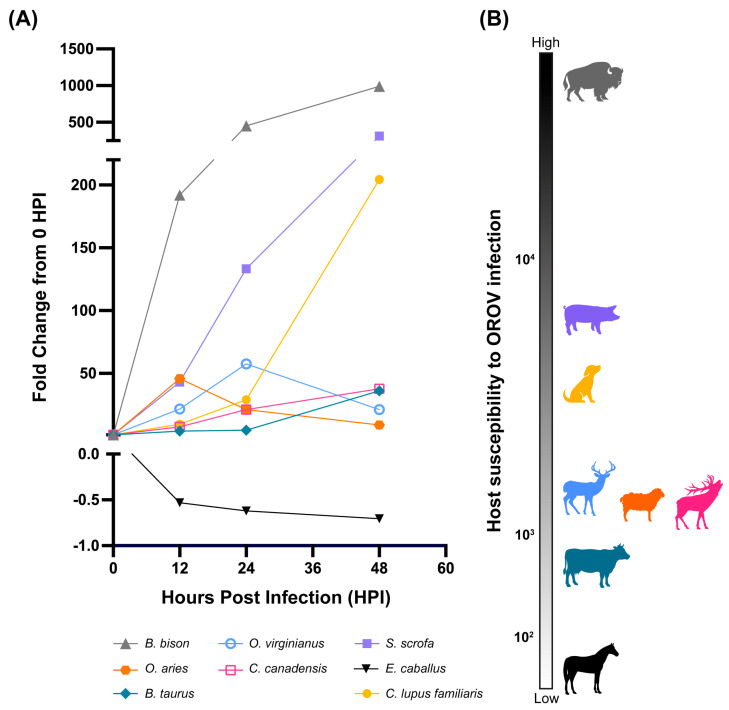
OROV^240023^ replication efficiency varies by host species. (**A**) Time course of fold change in viral titers by species. (**B**) Host species susceptibility for OROV^240023^ infection ranked from highest to lowest area under the curve (AUC) values for the fold change data. Mean AUC values are reported in Table 2.

To predict which species are most likely to be susceptible hosts for OROV^240023^, a between-species analysis of the fold change in viral titers by time was conducted (Figure 3A). The fold change in viral titers was significantly different by species and time (RM 2-Way ANOVA F (9.2, 21.0) = 25.1, *p* < 0.0001). The area under each curve (AUC) was calculated for three replicates by species and ranked to determine the overall risk for OROV^240023^ infection independent of viral titers at specific timepoints which could be influenced by the different cell types and not accurately reflect differences in host susceptibility (Figure 3B). The species with the highest mean AUC was *B. bison* followed by *S. scrofa* and *C. lupus familiaris*, but the mean AUCs were 70% and 86% less than that of *B. bison*, respectively (Table 2). The remaining species mean AUCs were all at least 93% less than that of *B. bison* (Table 2).

## 4. Discussion

Numerous orthobunyaviruses are known to cause severe disease in livestock worldwide, including well-known Cache Valley and Schmallenberg viruses [22,23,24,25] but also lesser-known Akabane [26,27,28], Aino [29,30], Shuni [31,32], Peaton [33], and Shamonda viruses [34,35]. Like OROV, many orthobunyaviruses are also transmitted by *Culicoides* spp. biting midges [15,27,35,36,37,38]. While OROV has not been associated with disease in livestock animals, its genetic and vector similarities with other orthobunyaviruses combined with antibody evidence suggest that susceptible animal reservoir species for OROV may exist. Elucidating the host range of OROV will clarify the sylvatic cycle, provide sentinel species to use for disease surveillance, and identify any livestock, wildlife, and domestic species at risk of disease.

Antibody evidence suggests that beyond sloths and non-human primates, dogs, cows, and sheep become exposed to OROV and therefore could be susceptible hosts for OROV [18,39]. Using cells derived from dog, cow, and sheep kidney cells, we found that OROV replicates in all three cell lines, further supporting these species as possible hosts. Additionally, we evaluated the infectability of cells from other animal species that are important representatives of North American livestock and wildlife populations and found evidence that bison, pigs, white-tailed deer, and elk should also be considered as potential OROV hosts. American bison, white-tailed deer, and elk are not native to and are not widely distributed across South America; therefore, these species are unlikely to be included in serological surveys from Brazil [40]. However, OROV antibodies have been found in Brazil in 0.4% of sheep [18,39], 10% of dogs and 7.5% of cows [18]. It should be noted that during the same period, Dias et al. 2022 did not detect OROV RNA in blood collected from febrile patients, 595 domestic mammals, and 215 wild mammals from the same urban and peri-urban locations as the serosurvey [41]. In Brazil, OROV-seropositive water buffalo (*Bubalus bubalis*), another member of the *Bovidae* family, have also been documented [42,43]. Water buffalo-derived cells were not included in the current study due to lack of availability.

Serological surveys have failed to find antibodies in equine species, including horses, donkeys, and mules [39,41] and pigs [18], although only 5 pigs were tested while 410 horses were tested (Pauvoild-Correa et al. tested 375 horses [18] and Dias et al. 2024 tested 35 horses [18]). We infected EqKd cells (equine kidney) and SK-RST (porcine kidney) cells and found robust replication in the SK-RST cells, but poor infection in the EqKd cells. Low infection in the equine cells combined with the negative serological surveys could mean that horses are not competent hosts for OROV. However, infection refraction in EqKd cells could also be due to tissue-specific tropism and not overall host incompetence. Tissue-specific tropism within horses has been documented for other viruses, such as equine influenza virus (EIV), which replicates efficiently in equine respiratory tissues but poorly in EqKd cells [44]. To understand if our results are generalizable, additional equine cells and tissues should be considered, such as neurons [45], trophoblasts [46], and leukocytes [47], which are susceptible to OROV infection in humans. Additionally, the OROV used to initiate the cell culture infections was passaged three times in Vero cells and contains two non-synonymous mutations in the L and M segments. These cell culture adaptation mutations could influence infectivity of other mammalian cell types. However, as OROV replicated to varying levels in the different cell types, it is unlikely that viral fitness increased due to the three passages in Vero cells.

A recent systematic review and meta-analysis found an urgent need to increase surveillance for animal reservoirs of OROV [20]. While virus infection of primary and immortalized cell lines can identify potential hosts, cellular permissibility for viral infection does not equate to organismal-level susceptibility. To confirm host status, in vivo studies are needed to evaluate viral replication dynamics, pathogenesis, and immune response at the organismal level. The current study provides candidate animal hosts, most notably bison, pigs, and dogs, for further evaluation in targeted field surveillance and experimental infection studies. Identifying the reservoir hosts of OROV will provide veterinarians, livestock producers, and wildlife managers with essential information to implement a targeted, host-specific disease surveillance program should OROV emerge into additional countries, like the United States and Mexico.

## Figures and Tables

**Table 1:** Species, tissue type, origin information, and media requirements for cells used in OROV infections.

**Cell Type**	**Species**	**Tissue**	**Origin**	**Base Media**	**Serum (%)**
WtLg	*Odocoileus virginianus*	Primary Lung	Wyoming USA 1989	M199	20
ELg	*Cervus canadensis*	Primary Lung	Wyoming USA 2000	M199	20
BKd	*Bos taurus*	Primary Kidney	Nebraska USA 2001	M199	20
BsKd	*Bison bison*	Primary Kidney	Wyoming USA 1991	M199	20
OEK	*Ovis aries*	Primary Kidney	Colorado USA 2001	M199	20
EqKd	*Equus caballus*	Primary Kidney	UNK	M199	20
MDCK	*Canis lupus familiaris*	Kidney	ATCC	eMEM	10
SK-RST	*Sus scrofa*	Kidney	ATCC	eMEM	10
Vero MARU	*Chlorocebus sabaeus*	Kidney	MARU 1973	M199	10

**Table 2:** Repeated-measures ANOVA F statistics and area under the curve (AUC) by species for Figure 1 and Figure 2.

**Species**	**Infectious Virus RM-ANOVA F Statistic** **(df)**	***p*** **-Value**	**RNA RM-ANOVA F Statistic** **(df)**	***p*** **-Value**	**Mean AUC** **PFU/mL ∗ hr (SE)**
*O. virginianus* (white-tailed deer)	105.4 (1.1, 2.2)	0.007	9.4 (1.0, 2.0)	0.09	1556.0 (186.6)
*C. canadensis* (elk)	22.0 (1.4, 2.8)	0.02	3.4 (1.3, 2.5)	0.19	930.9 (127.3)
*B. bison* (American bison)	118.5 (1.8, 3.6)	0.0006	246.6 (1.08, 2.2)	0.003	22,298 (4719.0)
*S. scrofa* (pig)	1569.0 (1.1, 2.2)	0.0004	9.6 (1.0, 2.0)	0.09	6662 (1748.0)
*O. aries* (sheep)	3.0 (1.1, 2.1)	0.2	2.0 (1.9, 3.9)	0.25	1043 (330.6)
*E. caballus* (horse)	33.0 (1.4, 2.9)	0.01	9.7 (1.1, 2.2)	0.08	27.8 (1.6)
*B. taurus* (cow)	16.7 (1.0, 2.0)	0.05	11.43 (1.0, 2.1)	0.07	573.6 (198.7)
*C. lupus familiaris* (dog)	112.5 (1.0, 2.0)	0.009	54.1 (1.0, 2.0)	0.02	3095.0 (879.2)

## Data Availability

All data generated during this study are included in this published article.

## Abbreviations

The following abbreviations are used in this manuscript:

OROV	Oropouche virus
HPI	Hours post infection
PFU	Plaque-forming unit
AUC	Area under the curve
USDA	United States Department of Agriculture
ICCL	Insectary and Cell Culture Laboratory
ABADRU	Arthropod-Borne Animal Diseases Research Unit
WSVDL	Wyoming State Veterinary Diagnostic Laboratory
FBS	Fetal bovine serum
MARU	Middle America Research Unit
ATCC	American Tissue Culture Collection
MOI	Multiplicity of infection
EIV	Equine Influenza virus
